# Spatial Variability of Heat-Related Mortality in Barcelona from 1992–2015: A Case Crossover Study Design

**DOI:** 10.3390/ijerph17072553

**Published:** 2020-04-08

**Authors:** Vijendra Ingole, Marc Marí-Dell’Olmo, Anna Deluca, Marcos Quijal, Carme Borrell, Maica Rodríguez-Sanz, Hicham Achebak, Dirk Lauwaet, Joan Gilabert, Peninah Murage, Shakoor Hajat, Xavier Basagaña, Joan Ballester

**Affiliations:** 1 Barcelona Institute for Global Health (ISGlobal), Barcelona 08003, Spain; vijendra.ingole@isglobal.org (V.I.);; 2 Climate and Health Program (CLIMA), Barcelona Institute for Global Health (ISGlobal), Barcelona 08003, Spain; 3 Agència de Salut Pública de Barcelona (ASPB), Barcelona 08023, Spain; 4 Biomedical Research Institute Sant Pau (IIB Sant Pau), Barcelona 08041, Spain; 5 Centro de Investigación Biomédica en Red de Epidemiología y Salud Pública (CIBERESP), Madrid 28029, Spain; 6 Universitat Pompeu Fabra (UPF), Barcelona 08003, Spain; 7 Centre for Demographic Studies (CED), Autonomous University of Barcelona, Barcelona 08193, Spain; 8 Environmental Modelling Department, Flemish Institute for Technological Research (VITO), Mol 2400, Belgium; 9 PCOT, Cartographic and Geological Institute of Catalonia (ICGC), Barcelona 08038, Spain; 10 Public Health, Environments and Society, London School of Hygiene and Tropical Medicine, London WC1H 9SH, UK

**Keywords:** heat-related mortality, UrbClim, spatial analysis, urban heat island effect, summer extreme heat

## Abstract

Numerous studies have demonstrated the relationship between summer temperatures and increased heat-related deaths. Epidemiological analyses of the health effects of climate exposures usually rely on observations from the nearest weather station to assess exposure-response associations for geographically diverse populations. Urban climate models provide high-resolution spatial data that may potentially improve exposure estimates, but to date, they have not been extensively applied in epidemiological research. We investigated temperature-mortality relationships in the city of Barcelona, and whether estimates vary among districts. We considered georeferenced individual (natural) mortality data during the summer months (June–September) for the period 1992–2015. We extracted daily summer mean temperatures from a 100-m resolution simulation of the urban climate model (UrbClim). Summer hot days (above percentile 70) and reference (below percentile 30) temperatures were compared by using a conditional logistic regression model in a case crossover study design applied to all districts of Barcelona. Relative Risks (RR), and 95% Confidence Intervals (CI), of all-cause (natural) mortality and summer temperature were calculated for several population subgroups (age, sex and education level by districts). Hot days were associated with an increased risk of death (RR = 1.13; 95% CI = 1.10–1.16) and were significant in all population subgroups compared to the non-hot days. The risk ratio was higher among women (RR = 1.16; 95% CI= 1.12–1.21) and the elderly (RR = 1.18; 95% CI = 1.13–1.22). Individuals with primary education had similar risk (RR = 1.13; 95% CI = 1.08–1.18) than those without education (RR = 1.10; 95% CI= 1.05–1.15). Moreover, 6 out of 10 districts showed statistically significant associations, varying the risk ratio between 1.12 (95% CI = 1.03–1.21) in Sants-Montjuïc and 1.25 (95% CI = 1.14–1.38) in Sant Andreu. Findings identified vulnerable districts and suggested new insights to public health policy makers on how to develop district-specific strategies to reduce risks.

## 1. Introduction

Global temperatures and heat wave frequency increases are projected to become more frequent and intense over the 21st century [1,2,3]. Epidemiological studies have analyzed the link between exposure to high temperature with adverse effects on mortality and morbidity [4]. The research on extreme temperature and mortality suggests that a wide range of individual and area level characteristics may affect susceptibility to heat, such as income, poverty, education level, greenspace, age and sex [5,6]. Elderly men and women are the most vulnerable groups; therefore, age and sex play a very important role; furthermore, there are other factors that modify the temperature related mortality relation, e.g., race, access to air conditioning, level of urbanization or cost of living and socio-economic status [7]. The urban heat island (UHI) is a well-known phenomenon in which temperature of an urban area is higher than the surrounding areas. The factors that cause UHI includes greenspace, impermeable space, surface roughness, albedo and emissivity [8].

Over roughly the past two to three decades, researchers have started examining the spatial distribution of heat-related mortality within urban areas. At the local scale, locations with higher morbidity and mortality rates during heat events have been associated with low socio-economic areas [9,10,11]. The majority of the heat-health research studies use the meteorological data from one or very few outdoor weather stations to estimate personal exposure, as these data are easily accessible, while indoor temperature data or personal exposure data are hard to find. However, exposure risk assessment requires consideration of all microenvironments where humans spend most of the time [12]. The weather-related mortality associations in a city using exposure variables from one local weather station or the average from a network of sites could induces exposure measurement error or biases in the estimates [13]. More recently, satellite-measured land surface temperature (LST) has been used to identify the temperature variations at a high spatial resolution [14]. However, LST cannot serve as a proper proxy for the daily mean temperature mainly due to their sparse temporal coverage (one measurement per day) [15]. Numerous studies have spatially interpolated temperature data from multiple measurement sites, but this is often limited by the sparse distribution of weather stations that cannot accurately measure temperature variations within a study area [13].

Recently, an urban climate model (UrbClim) was designed to model the urban influence on large-scale meteorological conditions at a resolution of a 100 m [16,17]. Epidemiological research assessments of climatic exposures could potentially benefit from using spatially explicit UrbClim model data, particularly in urban areas with few weather observation stations or in cities where the weather reported at the observation station is not actual representative of the local weather conditions experienced by the population.

Previous research in Barcelona metropolitan areas found that individual and neighborhood level factors were associated with high mortality during summer periods. These factors include old buildings, manual workers and perception of surrounding greenness, which were strongly associated with heat-related mortality [18]. In order to design spatially targeted interventions, a better understanding of heat risk is important for effective decision support [19,20]. Therefore, the present study aims to determine the spatial variability at district level in heat related mortality and to characterize the heat island effect by age, sex and education level in Barcelona in the years 1992–2015.

## 2. Materials and Methods

### 2.1. Study Population and Characteristics

Barcelona, the second largest city in Spain (1,650,358 inhabitants in January 2019 by Municipal Population Register of Barcelona City Council), is located on the northeastern coast of the Iberian Peninsula. Barcelona has 10 districts, and a map of the 10 districts is provided in the Supplementary Materials. Barcelona’s climate is Coastal Mediterranean. Winters are short and mild, while summers are long and hot. The average annual temperature is between 15 °C and 18 °C.

The mortality data were collected from the mortality registry of Barcelona, which collects information from the mortality registry of the Department of Health of Catalonia and from the municipal population registry. The dataset includes individual all-cause (natural) mortality records for the residents in the city of Barcelona. The individual mortality data were collected for the period of 1 January 1992 to 31 December 2015. Only the summer months’ deaths were considered in the analysis (1 June–30 September). The detailed information of each individual death includes date of death (day, month and year), age (through date of birth) and sex (men and women). Then, as a measure of socio-economic position, we used education level and grouped them into two categories (without studies and with primary education or higher), the data for which were obtained from the population register of the city, after excluding the deaths from the youngest age groups (<13 years old). Also, residence of deaths was georeferenced, both address (latitude and longitude) and district of residence. Residents of Barcelona who died outside the city were excluded from the analysis.

### 2.2. Urban Climate Model (UrbClim)

**Figure 1:**
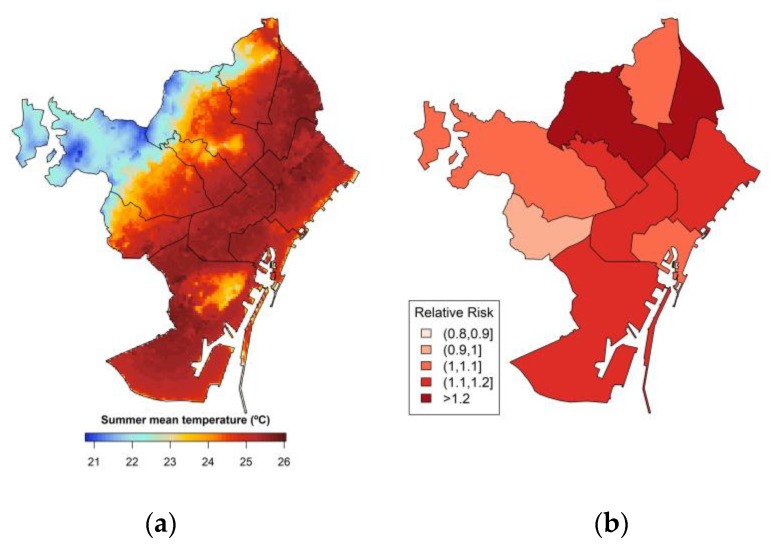
(**a**) Map of summer mean (1 June–30 September) temperature in (°C) and (**b**) relative risk map of all-cause mortality related to summer extreme temperatures by district during the study period 1992–2015 in Barcelona.

The UrbClim model was designed to simulate temperature and heat-stress at the city scale with a minimum amount of computation power [21]. This model is composed of a simple urban physics, coupled with a 3-D atmospheric boundary layer module. In the land surface scheme, urban terrain is represented as an impermeable slab with appropriate parameter values for albedo, emissivity and aerodynamic and thermal roughness length, and also accounting for anthropogenic heat fluxes [16]. UrbClim combines information about the urban structure (vegetation, soil sealing, typology, land use and land cover) through a simplified physics approach relevant at urban scales, and generates local climate data at very high spatial resolution of 100 m. The location and time were considered when climate data were extracted from the UrbClim model in order to merge with the georeferenced mortality dataset. The model has been previously validated in several validation campaigns, among which one has focused on the agglomeration of Barcelona [16,21,22]. Using the UrbClim model, daily urban climate data have been composed for all summer periods (1 June–30 September) of the years 1992–2015 (Figure 1a). The UrbClim model provided gridded daily climate data with a 100 m spatial resolution with a horizontal grid included in an area of 40 × 40 km, which covers the entire geographical area of the Barcelona metropolitan area, including the neighboring highly populated cities. This raw output is subsequently converted to daily minimum, mean and maximum temperature values along with location specific 5th to 95th percentile for the daily mean summer temperature. The yearly summer mean temperature trend is increasing as expected in recent years.

### 2.3. Exposure Definition and Lags Structures

The exposure data were extracted using time and location information (geographical coordinates of residence address of the deceased and date of death) and calculated daily mean summer temperature percentiles (5th to 95th) for each death case and its controls. The exposure variable group was defined based on the 70th–100th percentile (preliminary analysis results showed adverse health effects at 70th percentile and above) of summer mean temperature and reference group was 0–30th percentile (minimum mortality percentile) of summer mean temperature. We categorized days in the 70th percentile and above as hot days and the days between the 0 to 30th percentile as a non-hot day. We have used the daily mean temperature because it is a better predictor of temperature-mortality relationships when compared with either maximum or minimum temperature [23]. We fitted models of the average of daily mean summer temperature over the same day and three days before the day of death (lags 0–3 days) in order to identify heat effects that are mainly immediate and generally more delayed up to three days.

### 2.4. Statistical Method/Analysis

We used a time-stratified case-crossover design, commonly used for analyzing the effects of short-term exposures [24]. Daily summer mean temperature on the day of death (case day) and, as relevant, on the preceding days, is compared with the temperature on control days on which the death did not occur. The case-crossover methods naturally control for potential confounding factors that are time-invariant or that vary slowly over time, for example, ethnicity, socio-economic status, smoking and healthcare. We used control days on the same day of the week as the case day, to automatically adjust for day of the week, and in the same calendar month to avoid the so-called overlap bias [25]. In the case-crossover study design, each death is a ‘case’ and each individual act as his or her own control. This method was initially developed to avoid selection bias in controls, and has since then been applied to the study of mortality risks, mainly related to air pollution and temperature. Exposure to ambient temperatures for a given case (i.e., date of death) is compared with the exposure at several control cases (i.e., reference days before or after the event) [24,25]. Using the conditional logistic regression model, the relative risk (RR) of all-cause mortality related to summer extreme temperatures was estimated with spatially referenced temperature and mortality data. Firstly, the RR by sex, age and education groups for the citywide; secondly, the RR by district and finally, the analysis of RR by district was conducted separately for population sub-groups by age, sex and education. The map of study area (10 district of Barcelona) with respective names is given in the Supplementary Materials.

## 3. Results

We collected mortality data by age, sex and education for the periods of 1 June–30 September. The dataset included 105,559 all-cause mortality (48.84% among men and 51.16% among women) given in Table 1. The characteristics of the temperature data of the UrbClim model presented in Table 2 and the 70th percentile of mean temperature (>27 °C) and 30th percentile (<24 °C) of mean temperature are compared.

### 3.1. Heat-Related Mortality in Barcelona

Citywide all-cause (natural) mortality related to summer extreme temperatures is significantly higher on short cumulative lags of 0 to 3 days (Table 3). For all-cause mortality, the relative risk increases by 13% (RR = 1.13; 95% CI = 1.10–1.16) on hot days compared to non-hot days, and was higher among women (RR = 1.16; 95% CI = 1.12–1.21), the elderly (RR = 1.18; 95% CI = 1.13–1.22) and in a residence with primary education or higher (RR = 1.13; 95% CI = 1.08–1.18).

### 3.2. The Spatial Distribution of Heat-Related Mortality by District

The heat-mortality association results by districts shows 6 out 10 are at higher risk during the summer temperature (Figure 1b). We found a significantly high risk of mortality on hot days with 25% increase risk in Sant Adreu and 20% increase risk in Horta-Guinardó (Figure 1b). We did not find significant risk in the Les Corts district (RR = 0.94; 95% CI = 0.83–1.08) and found a low risk in three districts (Nou-Barris, Cuitat Vella and Sarrià-Sant Gervasi) (Table 4 and Figure 1b), respectively.

### 3.3. The Spatial Distribution of Heat-Related Mortality Results Stratified by Age, Sex and Education

**Figure 2:**
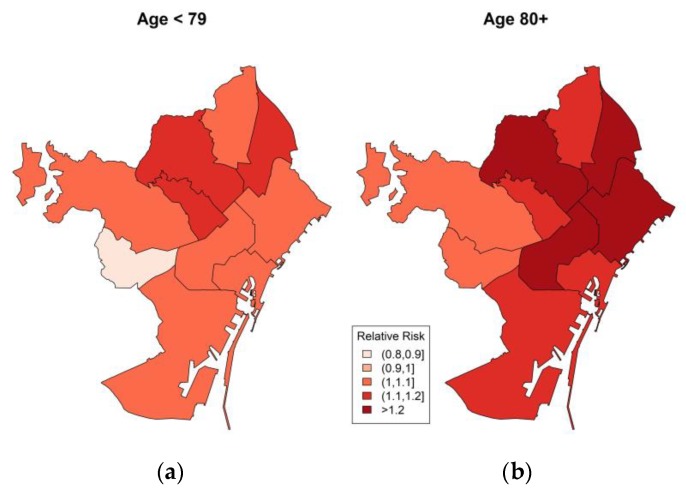
Relative risk of all-cause mortality related to summer extreme temperatures by district during the study period 1992–2015 in Barcelona in (**a**) age group less than 79 and (**b**) age group 80 and older.

The results for districts stratified by age, sex and education are presented in the Supplementary Material (Tables S1, S2 and S3). In Figure 2, the relative risk maps by district shows non-significant results in the age group <79 years (Figure 2a), except the districts Sant Andreu (RR = 1.19; 95% CI = 1.04–1.37) and Horta-Guinardó (RR = 1.17; 95% CI = 1.04–1.32). The elderly ≥80 years shows (Figure 2b) significant relative risk in all the districts, excluding Les Corts (RR = 1.01; 95% CI = 0.85–1.20). The highest relative risks among elderly (≥80 years) were observed in district Sant Andreu (RR = 1.32; 95% CI = 1.15–1.52), Horta-Guinardó (RR = 1.23; 95% CI = 1.10–1.38) and Sant Martí (RR = 1.23; 95% CI = 1.10–1.37).

**Figure 3:**
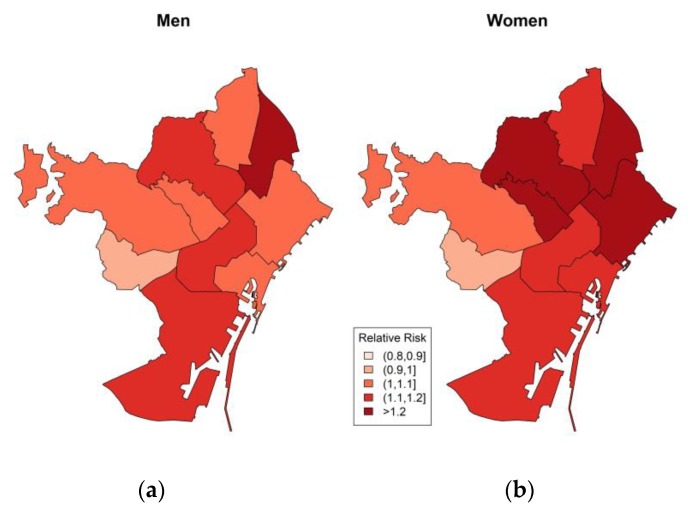
Relative risk of all-cause mortality related to summer extreme temperatures by district during the study period 1992–2015 in Barcelona in (**a**) men and (**b**) women.

Figure 3 presents the relative risk by districts in men (Figure 3a) and women (Figure 3b). The relative risk among men was statistically significant in the district Eixample (RR = 1.15; 95% CI = 1.05–1.26), Horta-Guinardó (RR = 1.17; 95% CI = 1.04–1.31) and even higher in Sant Andreu (RR = 1.28; 95% CI = 1.12–1.47). The relative risks among women was higher in all district except Les Corts, Cuitat Vella and Sarrià-Sant Gervasi. The district Gràcia, Horta-Guinardó, Sant Marti and Sant Andreu are the most vulnerable districts for women.

**Figure 4:**
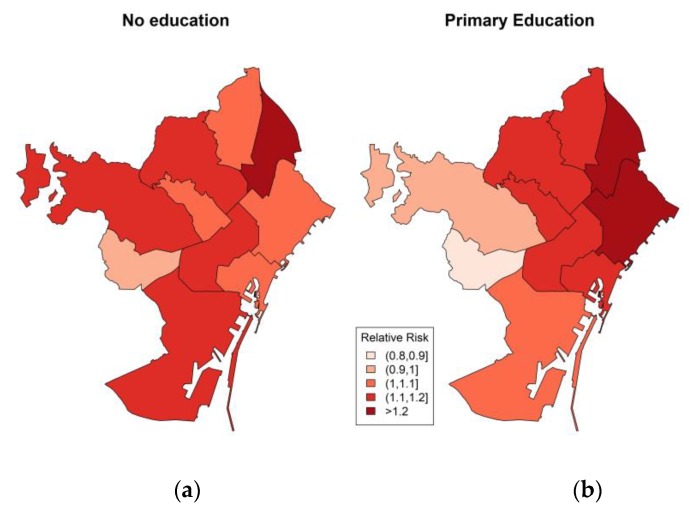
Relative risk of all-cause mortality related to summer extreme temperatures by district during the study period 1992–2015 in Barcelona with (**a**) no education and (**b**) with primary education.

The relative risk maps stratified by education level were obtained (Figure 4a,b). The population group with studies were more at risk in the districts Eixample, Sants-Montjuïc, Horta-Guinardó and Sant Andreu (RR = 1.21; 95% CI = 1.04–1.41). Similarly, the group with primary or higher education was statistically significant and particularly high in 3 districts (Eixample, Sant Andreu and Sant Martí) (detailed results presented in Supplementary Table S3).

## 4. Discussion

### 4.1. Summary of Main Results

To our knowledge, this is the first study conducted using spatially referenced climate-mortality data and high-resolution urban climate data in Barcelona, Spain. We studied the heterogeneity of the effect of three consecutive hot days (during summer months 1 June to 30 September) on mortality in the Barcelona and identified several subpopulations vulnerable to heat. The thresholds for hot days during summer periods were in the 70th–100th percentile (showed in our preliminary analysis the adverse health effects at 70th percentile and above). The overall results of the analysis suggest that the elderly and women have a higher risk of mortality on hot days during summer periods. This is consistent with the other studies that were conducted in Barcelona and elsewhere [5,6,7,23,26,27,28]. The spatial analysis revealed significant spatial variation in the heat-related mortality at a district level. In our analysis, the spatial variability of heat-related mortality shows more than 50% of the districts are at higher risk. The northern part of city especially has shown a significantly higher risk of mortality during the summer high temperature. This result support our hypothesis that heat-related mortality is spatially variable within urban areas. The citywide and district-wise heat-related mortality risks by age and sex showed clearly that the elderly and women were more vulnerable groups, which supports previous findings in Barcelona and elsewhere [5,6,29,30].

### 4.2. Spatial Differences by Age, Sex and Education

The spatial distribution of the relative risk by age demonstrated that there is a 32% higher risk in the Sant Andreu district, and the highest risk is among all of the districts for the elderly population (Figure 2b). Additionally, the districts Sant Martí, Horta-Guinardó and Eixample also shows the strongest risk for the elderly. We found that Sant Andreu, Horta-Guinardó and Gràcia are at lower risk (19%, 17% and 10%) for the age group of <79 years compared to the elderly group; this suggests that younger individuals are potentially healthier and less sensitive to heat. Another reason could be the thermoregulatory mechanism in the elderly, combined with a diminished ability to detect changes in their body temperature, which may partially explain their increased susceptibility [27].

The spatial differences in risk by sex was higher among women in all districts except Les Corts (Figure 3b). The most vulnerable area for men was the central north parts of the city, whereas Sant Andreu district shows the highest risk at 28%. The differences in age and sex might be a result of differences in socioeconomic, cultural and health-related factors. The relative risk by education level demonstrated that both groups with education and no education are at higher risk and contrast other findings; however, similar findings have shown this in previous studies in Barcelona [5]. The research in Barcelona found that excess mortality during the summer of 2003 was observed in all educational groups, and risk was higher for women with low education or with less than primary education [6]. However, we have not stratified education group by sex due to the limited number of cases.

### 4.3. Socio-Economic and Built Environmental Factors

It is well known that the socio-demographic characteristics of heat vulnerability such as race, income and education are mediated by more downstream mechanisms of heat vulnerability, such as cultural and social isolation, poor housing or poverty [5,31]. We have run meta-regressions to explore the effects of modification of the heat-mortality association by socio-economic and built environmental factors at a neighborhood level, including air pollution, family income index, unemployment, housing thermal demand and green space. We did not identify any of these indicators as a key factor in the heat-related deaths in the meta regression analysis (method and results are not shown here). This does not show that green space is not favorable; however, it may be that there are many other variables that confound the signal, especially at city scale. We are continuing to investigate the relationship at a small area level analysis using a Bayesian statistical model framework. Previous findings in Barcelona show that heat-mortality association was greater at the census level and it was higher among the residents living in older buildings, manual workers and the individual’s perception of green space [18]. The sociodemographic predictors, in contrast, are more relevant to risk perceptions at very small scales [2]. Previous research studies in the US and Europe have identified sociodemographic indicators that have shown different relative vulnerabilities to high temperatures [32,33], although this has been found not to be the case for the UK or Europe [26,34], and similarly with our results. Another case-crossover study design in Massachusetts evaluated individual and area-level effect modifiers of the warm temperature-mortality association and found modification by low income area and population density, but not by green space [35].

## 5. Strengths and Limitations

Our analysis has several strengths. The UrbClim model provides spatially resolved climate data that may offer improved exposure estimates, but is has not been systematically evaluated for use in epidemiologic evaluations. Here, we attempt to examine the high precision spatially referenced mortality and UrbClim model data. Recent research, for example, shows that static (airport) location temperature data may underestimate the effect of temperature on mortality when compared with a higher resolution, satellite-derived, spatially continuous estimate of the same variables [36]. Future epidemiological research on assessments of climatic exposures could potentially benefit from using spatially explicit weather data, particularly in places with few weather observation stations or in cities where the weather reported at the observatory is not actually representative of the weather experienced by the people [37]. The data collected from one single weather station cannot represent the spatial differentiation and, hence, could lead to substantial exposure measurement errors. Most of the epidemiological studies on heat and health rely on climate observations from the nearest meteorological station to assess exposures for geographically diverse populations [37,38]. The meteorological observations from a sparse station network do not adequately represent the range of conditions experienced by the human population. The meteorological observations are influenced by surrounding land cover (e.g., forest, urban areas) [37] and may be drawn from instruments deployed for reasons other than estimating heat-health effects (e.g., at airport to support flights for landing and takeoff). In urban areas, the built environment and land use heterogeneity modifies the exchange of energy and moisture between the surface and atmosphere, creating local climate variability and the urban heat island [8].

There are a few limitations we faced in modelling the risk that merit discussion. Previous validation exercises have shown that UrbClim, coupled to reanalysis data, can sometimes show a little warm bias in the modeled air temperatures Therefore, it is important to recognize the specific limitations both in the analysis and the interpretation of results obtained by using model data [21,39]. The socio-demographic variables were derived from data available at a fixed point in time (the year 2012 until 2016). However, the underlying demographics data ordinances both changed over time, a process we were unable to capture using meta-regression approach. This introduces some uncertainty into the results, and future research should explore local-scale mortality patterns over both space and time using the Bayesian statistical approach. Another limitation should be acknowledging that when we mapped the vulnerability of the population groups at the neighborhood level at very small scale, we did not find any significant differences among the geographical areas, and therefore, the unit of the analysis has been changed to the (bigger) district level in order to increase the statistical power of the analyses. The reason could be the Modifiable Areal Unit Problem (MAUP), which is known to be a potential source of error that can affect results. Therefore, the areas were aggregated at bigger scale; however, it may hide or bias the true spatial pattern [40]. Recently, Barcelona has changed a lot and there has been an especially large immigration of young population since 2000. The migration in Barcelona is an additional factor influencing the mortality trend [41]. We did not incorporate air quality data into this study but encourage future study of the interactive effects of heat and air quality on summertime mortality as well as the potential for differential mortality over space as a result of local-scale air quality variability.

## 6. Conclusions

In this research, we identified small scale spatial variability in heat-related mortality within Barcelona, Spain, over the period of 24 years. UrbClim model offers spatially explicit meteorological assessments by providing continuous weather data across space and time, which is not possible with a finite number of point-based weather stations. Furthermore, research is needed, and the evidence suggested that the spatially referenced meteorological data could possibly reduce exposure measurement error in epidemiological studies on heat and health [37]. In cities where high summer temperatures lead to elevated mortality rates, there is statistically significant spatial variability in the sensitivity to heat. Mortality records from 1992 to 2015 spanning 24 years show that residents of certain districts in the city have been at greater risk of dying when extreme heat occurs. To the best of our knowledge, this study is one of the first to document such district level variability in risk using long-term health records. The identified areas can be targeted to develop and improve strategies to create a better understanding of where people have higher risk and to design and evaluate interventions, and to target community engagement and outreach activities to reduce the health burden of heat.

## Figures and Tables

**Table 1:** Characteristics of deaths in summer (1 June–30 September) during the study period 1992–2015 in Barcelona.

Variables	*n*	%
Sex		
Men	51,556	48.84
Women	54,003	51.16
Total	105,559	100
Age		
<79	48,788	46.22
≥80	56,771	53.78
Total	105,559	100
Education *		
No Education	32,894	46.92
With Education	37,216	53.08
Total	70,110	100

* In this category, the individuals aged 13 years and older were excluded.

**Table 2:** Characteristics of temperature, in °C, in summer (1 June–30 September) estimated by UrbClim Model data during the study period 1992–2015 in Barcelona.

Temperature Variables	Mean	Std. Dev.	Minimum	Maximum
Mean Temperature	25.25	2.90	13.24	34.05
Maximum Temperature	28.39	3.23	14.51	40.52
Minimum Temperature	22.05	2.99	10.00	30.71
Percentile 70th of mean temperature	26.90	4.44	22.63	27.63
Percentile 30th of mean temperature	23.78	0.452	19.57	24.54

Std. Dev. —standard deviation.

**Table 3:** Citywide relative risk of all-cause mortality related to summer extreme temperatures by sex, age and education during the study period 1992–2015 in Barcelona.

Characteristics	Lag 0–3 days	
	Relative Risk	95% CI
All-cause mortality	1.13	1.10–1.16
Sex		
Men	1.10	1.06–1.14
Women	1.16	1.12–1.21
Age		
<79	1.08	1.04–1.12
≥80	1.18	1.13–1.22
Education *		
No Education	1.10	1.05–1.15
With Education	1.13	1.08–1.18

* In this category, the individuals aged 13 years and older were excluded. CI—confidence interval.

**Table 4:** Relative risk of all-cause mortality related to summer extreme temperatures by district during the study period 1992–2015 in Barcelona.

District Name	Lag 0–3 days		
	Relative Risk	95% CI	
Cuitat Vella	1.09	0.99	1.21
Eixample	1.17	1.10	1.24
Sants-Montjuïc	1.12	1.03	1.21
Les Corts	0.95	0.83	1.08
Sarrià-Sant Gervasi	1.04	0.95	1.15
Gràcia	1.14	1.04	1.26
Horta-Guinardó	1.20	1.10	1.31
Nou-Barris	1.08	0.99	1.18
Sant Andreu	1.25	1.14	1.38
Sant Martí	1.14	1.06	1.23

## Supplementary Materials

The following are available online at https://www.mdpi.com/1660-4601/17/7/2553/s1, Table S1: District wise relative risk for all-cause mortality (lag 0–3 days) by age group during the study period 1992–2015 in Barcelona, Table S2: District wise relative risk for all-cause mortality (lag 0–3 days) by sex group during the study period 1992–2015 in Barcelona, Table S3: District wise relative risk for all-cause mortality (lag 0–3 days) by education group during the study period 1992–2015 in Barcelona, **Figure S1**: Barcelona district boundary map.
