# Supplementary material for: Spatial Variability of Heat-Related Mortality in Barcelona from 1992–2015: A Case Crossover Study Design
Source: Int J Environ Res Public Health. 2020 Apr 8;17(7):2553. doi: 10.3390/ijerph17072553 (PMC7177772; doi:10.3390/ijerph17072553)
Supplement: Supplementary file 1 [file ijerph-17-02553-s001.zip › ijerph-751560-supplementary table .pdf]

Table S1. District wise relative risk for all-cause mortality (lag 0–3 days) by age group during the study period 1992–2015 in Barcelona.

District Name	Age <79		Age >80	
	Relative Risk	95% CI	Relative Risk	95% CI
Cuitat Vella	1.06	0.92–1.22	1.13	0.98–1.31
Eixample	1.09	0.99–1.20	1.22	1.13–1.32
Sants-Montjuïc	1.08	0.97–1.22	1.15	1.03–1.29
Les Corts	0.87	0.72–1.06	1.01	0.85–1.20
Sarrià-Sant Gervasi	1.07	0.92–1.24	1.03	0.91–1.16
Gràcia	1.10	0.95–1.27	1.18	1.04–1.33
Horta-Guinardó	1.17	1.04–1.32	1.23	1.10–1.38
Nou-Barris	1.01	0.90–1.14	1.16	1.02–1.32
Sant Andreu	1.19	1.04–1.37	1.32	1.15–1.52
Sant Martí	1.06	0.95–1.18	1.23	1.10–1.37

Table S2. District wise relative risk for all-cause mortality (lag 0–3 days) by sex group during the study period 1992–2015 in Barcelona.

District Name	Men		Women	
	Relative Risk	95% CI	Relative Risk	95% CI
Cuitat Vella	1.07	0.93–1.23	1.11	0.96–1.29
Eixample	1.15	1.05–1.26	1.19	1.09–1.29
Sants-Montjuïc	1.10	0.98–1.23	1.14	1.01–1.28
Les Corts	0.97	0.81–1.17	0.92	0.77–1.11
Sarrià-Sant Gervasi	1.04	0.90–1.19	1.05	0.93–1.19
Gràcia	1.03	0.89–1.18	1.25	1.10–1.42
Horta-Guinardó	1.17	1.04–1.31	1.24	1.10–1.39
Nou-Barris	1.03	0.92–1.16	1.14	1.01–1.30
Sant Andreu	1.28	1.12–1.47	1.23	1.07–1.41
Sant Martí	1.07	0.96–1.19	1.22	1.09–1.36

Table S3. District wise relative risk for all-cause mortality (lag 0–3 days) by education group during the study period 1992–2015 in Barcelona.

District Name	No Education		With Education	
	Relative Risk	95% CI	Relative Risk	95% CI
Cuitat Vella	1.06	0.90–1.25	1.11	0.89–1.38
Eixample	1.18	1.06–1.30	1.16	1.05–1.28
Sants-Montjuïc	1.14	1.00–1.30	1.00	0.85–1.19
Les Corts	0.98	0.78–1.24	0.89	0.73–1.07
Sarrià-Sant Gervasi	1.19	0.98–1.43	0.98	0.87–1.11
Gràcia	1.08	0.92–1.26	1.10	0.94–1.28
Horta-Guinardó	1.19	1.04–1.37	1.15	0.98–1.36
Nou-Barris	1.08	0.93–1.25	1.13	0.92–1.38
Sant Andreu	1.21	1.04–1.41	1.27	1.04–1.55
Sant Martí	1.08	0.95–1.22	1.22	1.04–1.44
