# Supplementary figures and images for: Spatial Variability of Heat-Related Mortality in Barcelona from 1992–2015: A Case Crossover Study Design
Source: Int J Environ Res Public Health. 2020 Apr 8;17(7):2553. doi: 10.3390/ijerph17072553 (PMC7177772; doi:10.3390/ijerph17072553)

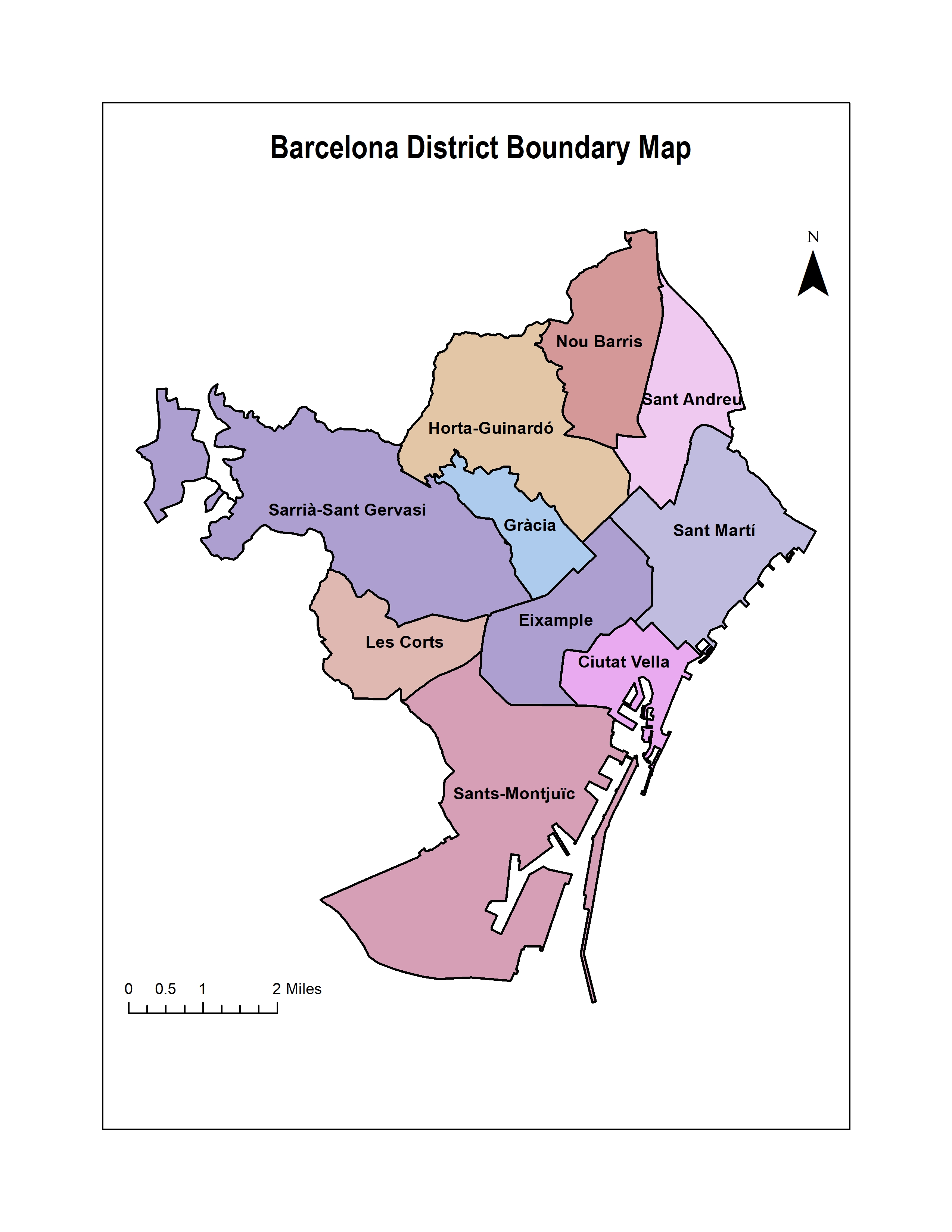

Supplement: Supplementary file 1 [file ijerph-17-02553-s001.zip › Figure S1. Barcelona district boundary map.jpg]
